# Anti-SARS-CoV-2 inactivated vaccine in patients with ANCA-associated vasculitis: Immunogenicity, safety, antibody decay and the booster dose

**DOI:** 10.1016/j.clinsp.2022.100150

**Published:** 2022-11-29

**Authors:** Rosa M.R. Pereira, Marilia A. Dagostin, Valeria F. Caparbo, Lucas P. Sales, Sandra G. Pasoto, Clovis A. Silva, Emily F.N. Yuki, Carla G.S. Saad, Ana C. Medeiros-Ribeiro, Leonard V.K. Kupa, Solange R.G. Fusco, Victor A.O. Martins, Carolina C.M.F. Martins, Carmen Valente Barbas, Samuel K. Shinjo, Nadia E. Aikawa, Eloisa Bonfa

**Affiliations:** a Rheumatology Division, Hospital das Clínicas, Faculdade de Medicina, Universidade de São Paulo (HCFMUSP), São Paulo, SP, Brazil; b Pediatric Rheumatology Unit, Instituto da Criança e do Adolescente, Hospital das Clínicas, Faculdade de Medicina, Universidade de São Paulo (HCFMUSP), São Paulo, Brazil; c Pulmonary Division, Hospital das Clínicas, Faculdade de Medicina, Universidade de São Paulo (HCFMUSP), São Paulo, SP, Brazil

**Keywords:** ANCA-associated vasculitis, Vaccine, SARS-CoV-2, Immunogenicity

## Abstract

**Objective:**

To evaluate inactivated CoronaVac prime vaccination, antibody decay, booster dose, and safety in ANCA-Associated Vasculitis (AAV) patients.

**Methods:**

Fifty-three AAV patients and 106 Controls (CG) received CoronaVac on days: D0 (first dose), D28(second dose), and D210 (booster dose, 32 AAV: 32 CG). The primary outcome was immunogenicity after the second vaccine dose (day 69) assessed by Seroconversion Rates (SC) of anti-SARS-CoV-2 S1/S2 IgG and Neutralizing Antibodies (NAb). Secondary outcomes were safety, immunogenicity (D28/D240), 6-months antibody decay (D210) and the booster dose response (D240).

**Results:**

At D69 SC (65.1% vs. 96.8%, p = 0.0001), GMT (21.3 UA/mL vs. 67.7 UA/mL, p < 0.001) and NAb- positivity (53.7% vs. 80.6%, p = 0.001) were moderate but lower in naïve-AAV patients than CG. Patients without SC used more often IS (93.3% vs. 53.3%, p = 0.015), mycophenolate mofetil (20% vs. 0%, p = 0.037) and prednisone (60.0% vs. 28.6%, p = 0.057) than seroconverted. NAb negativity in AAV patients was associated with prednisone treatment (57.9% vs. 18.2%, p = 0.015) and IS (84.2% vs. 55.0%, p = 0.046). Logistic regression analysis models showed that only prednisone was associated with lower seroconversion (OR = 0.2, 0,95% CI 0.05‒0.86, p = 0.030) and with lower NAb positivity (OR = 0.2, 0,95% CI 0.05‒0.88, p = 0.034). After six months (D69‒D210) a decrease in IgG positivity occurred in 32 AAV patients (15.7%, p = 0.074) and 32 CG (18.7%, p = 0.041). For the NAb positivity, the 6-month decrease was not significant (p = 0.114) whereas a major reduction occurred for CG (p < 0.001). A booster dose (D240) resulted in an increment in IgG-positivity (21.9%, p = 0.023) and NAb-positivity (34.4%, p = 0.006) in AAV patients. No moderate/severe adverse events attributable to the vaccine were observed.

**Conclusion:**

This study provides novel data on the excellent safety and moderate immunogenicity of CoronaVac in AAV patients. A six-month mild antibody waning was observed with a good response to the booster dose, although levels remained lower than CG (CoronavRheum-NCT04754698).

## Highlights

• This study provides evidence that CoronaVac, an inactivated virus vaccine against SARS-CoV-2, is safe and has a moderate immunogenicity in AAV.
• The authors identified that immunogenicity is negatively influenced by glucocorticoids.
• A mild antibody decay occurs in 6-months with a good response with a booster dose, although lower than health controls.

## Introduction

Coronavirus Disease 2019 (COVID-19) causes Severe Acute Respiratory Syndrome and the agent Coronavirus 2 (SARS-CoV-2), emerged in 2019 and has spread rapidly since then. The death toll of the pandemic is estimated to be millions and brought major damage not only in health-related issues but also in social and economic aspects across the globe.^1^^,^^2^ By the time of this submission, more than 460 million people have been infected with SARS-CoV-2 and nearly 6 million died from COVID-19 (WHO ‒ https://covid19.who.int/).

Pharmacological antiviral therapy for COVID-19 patients is scarce and not widely available, and therefore supportive care measures such as ventilation oxygenation and fluid management remain the standard of care.^3^ Consequently, mass vaccination is the most effective strategy for controlling the pandemic so far. In the past 18 months, several vaccines have been developed and commercialized in record time, with proven efficacy in phase III trials,^4^, ^5^, ^6^ including CoronaVac,^7^ an inactivated virus vaccine against SARS-CoV-2, with emergency use approval by the World Health Organization (WHO) in several most populated countries, including Brazil.

Although there are a number of papers evaluating the safety and efficacy of the COVID-19 vaccines in overall Autoimmune Rheumatic Diseases (ARD)^8^, ^9^, ^10^, ^11^ none focused specifically on rare diseases such as AAV. These individuals are the ones that theoretically have the greatest benefit from vaccination since their condition is frequently aggravated by renal and lung function impairment with a consequent increase in the risk of severe SARS-CoV-2 infection and death.^12^, ^13^, ^14^ It is not known if high immunosuppression would impact immunogenicity and the dynamics of 6-months antibody decay or booster dose. In addition, regarding safety, there is a concern if the level of disease activity would influence vaccine immunogenicity or else if the vaccine may trigger or aggravate systemic inflammation.

The CoronavRheum trial, a large Brazilian phase 4 trial in 910 adults with ARD showed that this vaccine has an overall moderate short-term immunogenicity although lower than the control group.^11^ Similarly, an mRNA COVID-19 vaccine induced reduced immune response in a cohort of global ARD patients compared to the control group, including a very small sample of AAV patients.^9^

Therefore, the aim of this study is to analyze CoronaVac safety, immunogenicity, antibody decay, and booster dose response in AAV patients and the Control Group (CG). The authors also evaluated the impact of disease activity and immunosuppressive treatment on the vaccine response of these patients.

## Materials and methods

This prospective controlled trial is within a large phase 4 study (CoronavRheum clinicaltrials.gov #NCT04754698) conducted at a single tertiary center in Sao Paulo (Brazil) that assessed immunogenicity and safety of the CoronaVac COVID-19 vaccine in a large sample of ARD patients.^11^ Data were collected and managed using REDCap electronic capture tools hosted at the studied Institution.^15^^,^^16^ The study was conducted according to the Declaration of Helsinki and local regulations and was approved by the local and national ethical committee (CAAE: 42566621.0.0000.0068). Written informed consent was obtained from all participants.

### Patients and controls

Consecutive naïve patients (COVID-19 seronegative) diagnosed with Granulomatosis with Polyangiitis (GPA), Eosinophilic Granulomatosis with Polyangiitis (EGPA), or Microscopic Polyangiitis (MPA) by the American College of Rheumatology Classification Criteria^17^^,^^18^ and the Chapel Hill Conference Classification^14^ aged ≥ 18 years old and with regular follow-up in the Vasculitis Outpatient Clinic were invited to participate in the study.

Subsequently, a CG of administrative hospital workers and their relatives was invited to participate. The two groups were age and sex-balanced (± 5 years) in a 2:1 ratio (2 controls: 1 patient) using an in-house Excel program (Microsoft 2018) for random selection of participants in each group. Autoimmune rheumatic disease diagnosis, use of immunosuppressants, or Human Immunodeficiency Virus (HIV) infection were exclusion criteria for CG, though other well-controlled diseases were allowed.

Exclusion criteria for all participants were: acute febrile condition or symptoms suggestive of COVID-19 at baseline, previous anaphylactic response to vaccine components, demyelinating disease, severe heart failure (class III or IV), history of having received blood transfusion ≤6-months before study entry, inactivated virus vaccine ≤ 14-days before study entry, history of live virus vaccine ≤4-weeks before study entry, individuals who did not consent to participate in the study, hospitalized patients, prior immunization with any SARS-CoV-2 vaccine, and pre-vaccination positive COVID-19 Serology (anti-S1/S2 IgG) and/or NAb for immunogenicity analysis of naïve-AAV patients.

### Vaccination and blood collection protocol

The study protocol consisted of five in-person visits that occurred on February 9^th^‒10^th^ 2021 (D0 ‒ first vaccine dose and blood collection), on March 9^th^‒10^th^ 2021 (D28 ‒ second vaccine dose and blood collection), on April 19^th^, 2021 (D69 – blood collection), on September 18^th^, 2021 (D210 – 3^rd^ vaccine dose and blood collection) and on October 19^th^, 2021 (D240 – blood collection) at the Hospital Convention Center (São Paulo, Brazil). The vaccination protocol for all participants included three doses of ready-to-use syringes loaded with the CoronaVac vaccine (Sinovac Life Sciences, Beijing, China, batch #20200412), consisting of 3 μg in 0.5 mL of β-propiolactone inactivated SARS-CoV-2 (resultant from the CN02 strain of SARS-CoV-2 grown in African green monkey kidney cells ‒ Vero 25 cells) with aluminum hydroxide as an adjuvant and applied in the deltoid muscle.

### Outcomes

The primary outcome was immunogenicity assessed by two co-primary endpoints: seroconversion of anti-SARS-CoV-2 S1/S2 IgG and the presence of NAb after the second vaccine dose (D69). Secondary outcomes were Geometric Mean Titers (GMT) and neutralizing activity at D69, immunogenicity parameters at D28, D210 (day of 3^rd^ dose) and D240 (30 days after 3^rd^ dose), and safety related to the vaccine doses. Additionally, factors associated with anti-SARS-Cov-2 IgG seroconversion and NAb positivity were evaluated.

To assess these outcomes, blood samples (20 mL) from all participants were obtained at all in-person visits.

### Anti-S1/S2 SARS-CoV-2 antibodies

Human IgG antibodies against the S1 and S2 proteins of SARS-CoV-2 were measured by chemiluminescent immunoassay as described previously.^14^ Seroconversion Rates (SC) were measured by positive serology (≥15.0 UA/mL) after vaccination, considering that only patients with pre-vaccination negative serology were included. GMT and 95% Confidence Intervals (95% CI) of these antibodies were also determined at all-time points, attributing the value of 1.9 UA/mL (half of the lower limit of quantification 3.8 UA/mL) to above lower levels (< 3.8 UA/mL). The Factor Increase in GMT (FI-GMT) is the ratio of the GMT after immunization to the GMT before immunization which identifies the increase in titers.

### Neutralizing antibodies (NAb)

The SARS-CoV-2 NAb analysis was performed according to manufacturer instructions using an sVNT Kit (GenScript, Piscataway, NJ, USA), as described previously.^11^ The samples were cataloged as “positive” (inhibition ≥30%) or “negative” (inhibition < 30%) according to the manufacturer.^19^ The frequency of seropositivity was calculated at all-time points. Medians (interquartile range) of the percentage of neutralizing activity were only measured for seropositive samples at all-time points.

### Vaccine adverse events

Adverse Events (AE) were carefully followed throughout the study. Patients and CG were advised to report any adverse events of the vaccine and they received on D0 and at all visits a standardized diary for local and systemic manifestations. AE severity was classified according to WHO criteria.^20^

In addition, incident COVID-19 cases were assessed in all subjects with instruction to notify any symptom associated or not with COVID-19 (by telephone, smartphone instant messaging, or email) and the disease was confirmed by RT-PCR test. Independent vaccine experts monitored the study regarding adverse events for data safety.

### Disease assessment

Demographic, clinical, and therapeutic data of the participants in the AAV group were recorded and compared regarding seroconversion and NAb positivity. The Birmingham Vasculitis Activity Score (BVAS) – version 3^21^ was assessed in all patients at baseline (at the most recent outpatient visit before vaccination) and after the second dose of the vaccine (at the next outpatient visit), to analyze the possible impact of disease activity in the vaccine immunogenicity, as well as the potential risk of the vaccine to trigger disease activity. Disease-related damage, expressed by the Vasculitis Damage Index (VDI), was also included in the analysis as a potential impact on seroconversion and production of Nab.^22^ Since the beginning of the trial there was no evidence-based information on the effect of immunosuppression on vaccine immunogenicity, the protocol did not include tapering or discontinuation of any treatment, and doses of prednisone and other immunosuppressants were maintained as directed by the disease status.

### Statistical analysis

Categorical variables were presented as numbers (percentage) and compared using the chi-square or Fisher's exact tests, as appropriate, and McNemar´s test for before and after comparisons in the same group. Continuous general data were presented as medians (minimum and maximum values) and compared using the Mann-Whitney test for intergroup comparisons and Wilcoxon signed rank test for before and after comparisons in the same group. Data regarding IgG titers were analyzed using Analysis of Variance (ANOVA) with repeated measures and two factors (two groups ‒ vasculitis versus CG ‒ at specified time points), followed by Bonferroni's multiple comparisons at neperian logarithm (ln)-transformed data. For patients with AAV, multivariate logistic regression analyses were performed using dependent variables SC or the presence of NAb at D69 (primary endpoints), and as independent variables with p < 0.05 in each univariate analysis. Statistical significance was defined as p < 0.05. All statistical analyses were performed using Statistical Package for the Social Sciences, version 20·0 (IBM-SPSS for Windows. 20.0. Chicago, IL, USA).

## Results

### Participants

**Fig. 1:**
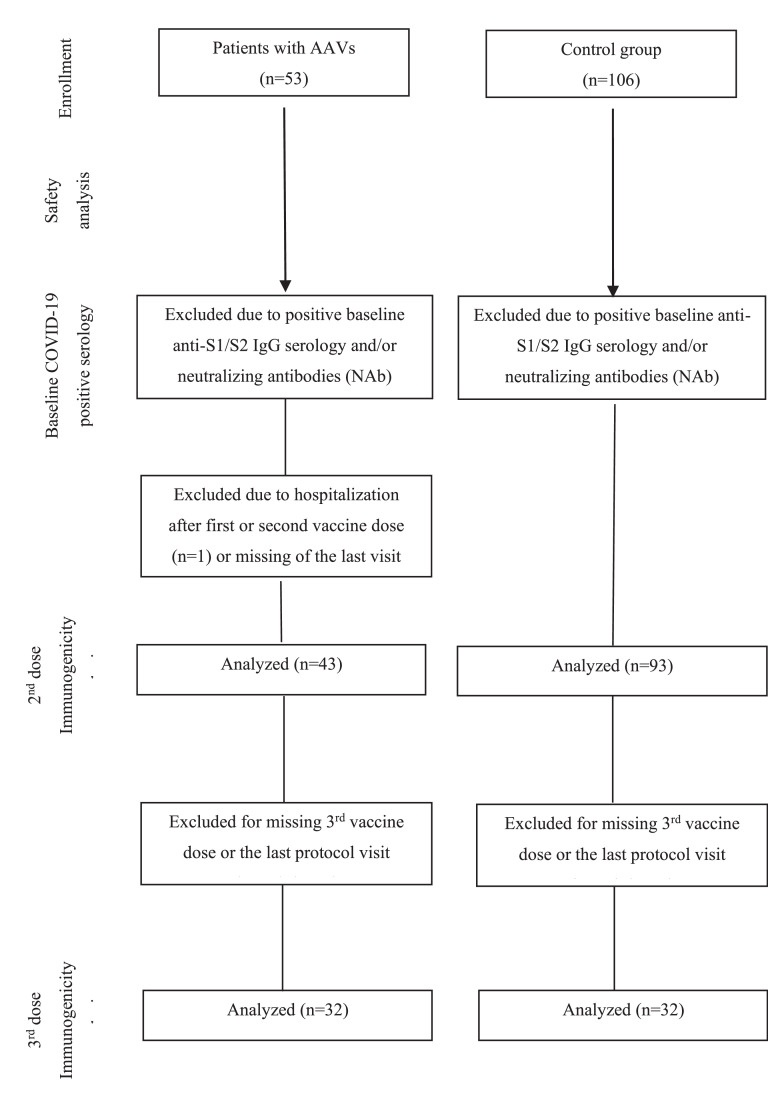
Flow-chart of the present study.

Fifty-three AAV patients (GPA [n = 36], EGPA [n = 10] and MPA [n = 7]) and CG (n = 106) were initially included in the study and received two doses of CoronaVac vaccine (Fig. 1). AAV patients and CG were balanced for sex and age. Patients with AAV had a median disease duration of 7 years (range: 1 to 31). Comorbidities were more frequent in the AAV group (83%, p = 0.0007), with systemic arterial hypertension (50.9%) being the most prevalent comorbidity. A total of 19 AAV patients were using prednisone (35.8%), 71.7% of patients were under immunosuppressive drugs, and 15.1% individuals were under rituximab treatment (Table 1).

**Table 1:** Baseline characteristics of ANCA-Associated Vasculitis (AAV) vasculitis patients and Controls (CG).

	AAV (n = 53)	CG (n = 106)	p-value
**Demographics**			
Current age, years	52 (24‒75)	52 (24‒78)	0.770
Age at diagnosis, years	42 (3‒71)	‒	‒
Disease duration, years	7 (1‒31)	‒	‒
Female sex	31 (58.5)	62 (58.5)	1.000
Caucasian race	33 (62.3)	52 (49)	0.131
BMI, Kg/m^2^	28.1 (18.4‒38.5)	26.6 (17.3‒39.1)	0.142
**Comorbidities**	44 (83)	59 (55.7)	**0.0007**
Systemic arterial hypertension	27 (50.9)	33 (31.1)	**0.023**
Diabetes mellitus	8 (15.1)	18 (17)	0.824
Dyslipidemia	10 (18.9)	11 (10.4)	0.144
Obesity	17 (66)	29 (52.8)	0.580
Chronic cardiomyopathy	3 (5.7)	4 (4.7)	0.687
Chronic renal disease	6 (11.3)	0	**0.001**
Current smoking	2 (3.8)	9 (8.5)	0.339
Chronic obstructive pulmonary disease	2 (3.8)	1 (1,9)	0.258
Asthma	7 (13.2)	4 (3.8)	**0.043**
Interstitial lung disease	3 (5.7)	0	**0.036**
Pulmonary hypertension	1 (1.9)	0	0.333
Hematologic disease	0	0	‒
Hepatic disease	1 (1.9)	0	0.333
Current cancer	1 (1.9)	0	0.333
Stroke	1 (1.9)	0	0.333
Current tuberculosis	0	0	‒
HIV	0	0	‒
**Vasculitis Score**			‒
BVAS	0 (0‒8)	‒	‒
VDI	3 (0‒9)	‒	‒
**Current therapy**			‒
Prednisone	19 (35.8)	**‒**	‒
Immunosuppressive drugs	38 (71.7)		‒
Methotrexate	14 (26.4)	**‒**	‒
Azathioprine	16 (30.2)	**‒**	‒
Mycophenolate mofetil	5 (9.4)	**‒**	‒
Cyclophosphamide	3 (5.7)	‒	‒
Leflunomide	1 (1.9)	‒	‒
Biologic therapy			‒
Rituximab	8 (15.1)	**‒**	‒

Results are expressed as median (minimum and maximum values) and n (%).

For immunogenicity analysis 10 AAV patients were excluded due to: positive pre-vaccination COVID-19 serology (n = 7), hospitalization (n = 1) and loss of follow-up (n = 2). Thirteen individuals from the naïve-CG were also excluded from the immunogenicity analysis due to positive pre-vaccination COVID-19 serology. The final group comprised 43 AAV patients and 93 CG (Fig. 1). These 43 AAV patients and 93 CG were further invited to participate in the decay and booster dose extension protocol. Only 32 patients completed the protocol and the authors subsequently randomly selected among the CG group 32 sex and age-balanced (± 5-years) in a 1:1 ratio (1 control: 1 patient) (Fig. 1).

### Immunogenicity

#### Anti-SARS-CoV-2 IgG antibodies production in 43 naïve-AAV and 93 naïve-CG groups at D69

The humoral response to CoronaVac is shown in Table 2. Analysis of the SARS-CoV-2 S1/S2 IgG response revealed a moderate seroconversion rate in patients with AAV six weeks (D69) after the second vaccine dose, although lower compared to CG (65.1% vs. 96.8%, p = 0.0001). GMT and FI-GMT were also lower in patients with AAV compared to CG (p < 0.001 and p < 0.001, respectively).

**Table 2:** Seroconversion rates and anti-SARS-CoV-2 S1/S2 IgG titers before and after the first and second doses of CoronaVac vaccination in ANCA-Associated Vasculitis (AAV) patients and Controls (CG).

	SC		GMT (AUmL^−1^)			FI-GMT	
	D28	D69	D0	D28	D69	D0 to D28	D0 to D69
**AAV (n = 43)**	5 (11.6)	28 (65.1)	2.2 (2.0‒2.4)	4.4 (3.2‒6.0)	21.3 (13.2‒34.5)	2.0 (1.53‒2.67)	9.8 (6.1‒15.8)
**CG (n = 93)**	32 (34.8)	90 (96.8)	2.4 (2.1‒2.6)	11.2 (8.4‒14.9)	67.7 (58.3‒78.6)	4.7 (3.7‒6.0)	28.7 (24.2‒34.1)
**p (AAV vs. CG)**	**0.0065**	**0.0001**	>0.999	**<0.001**	**<0.001**	**<0.001**	**<0.001**

SC is defined as post-vaccination titer ≥15 AU mL^−1^ by indirect ELISA, LIAISON SARS-CoV-2 S1/S2 IgG. Frequencies of SC are presented as number (%) and were compared using a two-sided chi-square test between AVV and CG at prespecified time points (D28 and D69). IgG antibody titers and FI-GMT are expressed as geometric means with 95% CI. Data regarding IgG titers were analyzed using ANOVA with repeated measures and two factors (two groups (vasculitis vs. CG) at three time points (D0, D28 and D69), followed by Bonferroni's multiple comparisons at ln-transformed data. The behavior of IgG titers was different for AAV and CG groups between D28 and D69: mean titers increased at each time point for AAV and CG (p < 0.001). FI-GMT values were compared using the Mann-Whitney *U*-test for intergroup comparisons in ln-transformed data at prespecified time points (D28 and D69). All analyses were two-sided.

#### NAb positivity in 41 naïve-AAV patients and 93 naïve-CG groups at D69

After the 2^nd^ vaccine dose, more than half of the AAV patients had positive NAb, a frequency lower than the CG group (53.7% vs. 80.6%, p = 0.001). Of note, the median of NAb activity was similar after the second dose (69.3 [47.2‒90.0] vs. 61.2 [46.3-80.1], p = 0.240) in patients and CG (Table 3).

**Table 3:** Frequency of neutralizing antibodies (NAb) and median percentage of neutralizing activity in positive cases, after the first and second doses of CoronaVac vaccination in ANCA-associated vasculitis (AAV) patients in comparison to controls (CG).

	D28		D69	
	Subjects with positive Nab, n (%)	Neutralizing activity (%)	Subjects with positive Nab, n (%)	Neutralizing activity (%)
		Median (interquartile range)		Median (interquartile range)
**AAV (n = 43)**	5 (11.6)	62.6 (53.2‒65.2)	22 (53.7)^a^	69.3 (47.2‒90.0)
**CG (n = 93)**	36 (40)	49.9 (35.9‒80.4)	75 (80.6)	61.2 (46.3‒80.1)
**p (AAV vs. CG)**	0.001	0.952	0.001	0.240

Frequencies of subjects with positive NAb are expressed as number (%). Positivity for NAb was defined as neutralizing activity ≥ 30% (cPass sVNT Kit). Data were compared using a two-sided Chi-Square test between AAV and CG at prespecified time points (D28 and D69). Percentage of neutralizing activity among subjects with positive NAb is expressed as median (IQR). Data were compared using a two-sided Mann-Whitney *U*-test for comparison between AAV and CG, at prespecified time points (D28 and D69).

a In D69, AAV patients n = 41 due to unavailability of two NAb samples.

#### Factors associated with seroconversion and NAb positivity among naïve-AAV patients at D69

Analyzing the possible impact of disease activity on the immunogenicity of the vaccine, the higher frequency of seroconversion rates at D69 in naïve AAV patients with BVAS activity score = 0 compared to those with BVAS > 0 did not reach statistical significance (74.2% vs. 41.7%, p = 0.074). GMT was comparable in both groups at D69 (Table 4). With regard to the possible influence of vaccine on disease activity there was no change in this parameter with similar BVAS levels at baseline and after the 2^nd^ vaccine dose (0.81 ± 1.64 vs. 1.07 ± 2.66, p = 0.71). GMT BVAS was also comparable at baseline and after the 2^nd^ vaccine dose (0.81 ± 1.64 vs. 1.07 ± 2.66, p = 0.71). There was no difference in the seroconversion rate among patients with AAV regarding baseline VDI (3.19 ± 0.35 with seroconversion vs. 3.17 ± 0.37 with no seroconversion, p = 0.975).

**Table 4:** Seroconversion rates at D69, anti-SARS-CoV-2 S1/S2 IgG titers comparing vasculitis activity score (BVAS = 0 vs. BVAS > 0) and frequency of NAb and median percentage of neutralizing activity after second dose (D69) of CoronaVac vaccination in ANCA-Associated Vasculitis (AAV) patients.

	SC	GMT (AU mL^−1^)
	D69	
**BVAS baseline = 0 (n = 31)**	23 (74.2)	25.5 (14.6‒44.5)
**BVAS baseline > 0 (n = 12)**	5 (41.7)	14.2 (4.7‒42.9)
**p (BVAS_baseline_ = 0 vs BVAS_baseline_ > 0)**	0.074	0.330
	**D69**	
	**Subjects with positive NAb, n (%)**	**Neutralizing activity (%) median (IQR)**
**BVAS baseline = 0 (n = 31)**	15 (48.4)	68.2 (44.1–89.7)
**BVAS baseline > 0 (n = 12)**	7 (58.3)	74.3 (63.3–90.6)
**p (BVAS_baseline_ = 0 vs BVAS_baseline_ > 0)**	0.736	0.587

Frequency of subjects with seroconversion is expressed in n (%). Titers of IgG antibodies are expressed in geometric means with 95% CI. BVAS, Birmingham Vasculitis Activity Score; SC, Seroconversion; Nab, Neutralizing antibodies; GMT, Geometric Mean Titers.

Regarding treatment, the frequencies of immunosuppressive drugs (93.3% vs. 53.3%, p = 0.015) and mycophenolate mofetil (20% vs. 0%, p = 0.037) were significantly higher in AAV patients without SC compared to those with SC, and a trend of more frequent prednisone use (60% vs. 28.6%, p = 0.057). Negative NAb in AAV patients was associated with more frequent use of prednisone (57.9% vs. 18.2%, p = 0.015) and immunosuppressive drugs (84.2% vs. 55.0%, p = 0.046) compared to those with positive NAb (Table 5). Eight patients who had received rituximab within 6 months before the first dose of the vaccine were considered to be on rituximab treatment. The median cumulative dose of rituximab was 4.5 g (minimum 2, maximum 8), and the median interval between the last rituximab cycle and the first vaccine dose was 2 months (minimum 0, maximum 5). Logistic regression analysis models showed that only the use of prednisone was associated with lower seroconversion (OR = 0.20, 95% CI 0.05‒0.86, p = 0.030) and lower NAb positivity (OR = 0.20, 95% CI 0.05‒0.88, p = 0.034).

**Table 5:** Baseline characteristics of AAV patients with and without Seroconversion (SC) for anti-SARS-CoV-2 S1/S2 IgG antibodies and with and without positivity of Neutralizing Antibodies (NAb) after two doses of CoronaVac vaccination (day 69).

	Vasculitis patients with SC (n = 28)	Vasculitis patients without SC (n = 15)	p-value	Vasculitis patients with NAb (n = 22)	Vasculitis patients without NAb (n = 19)	p-value
**Demographics**						
Current age, years per median (mn ± max)	52.6 ± 13.4	53.0 ± 12.5	0.926	54.1 ± 15.2	51.1 ± 10.8	0.479
Current age > 60 years	6 (21.4)	5 (33.3)	0.473	8 (36.4)	3 (15.8)	0.173
Female sex	17 (60.7)	8 (53.3)	0.750	14 (63.6)	11 (57.9)	0.757
Caucasian race	17 (60.7)	11 (73.3)	0.512	15 (68.2)	12 (63.1)	0.754
**Current therapy**						
Prednisone	8 (28.6)	9 (60)	0.057	4 (18.2)	11 (57.9)	**0.015**
Prednisone dose, mg	5 (1.7‒20)	10 (5‒40)	0.234	0 (0‒20)	1.7 (0‒40)	0.280
Prednisone *≥* 20 mg/day	2 (7.1)	4 (26.7)	0.161	3 (13.7)	2 (10.5)	1.000
Immunosuppressive drugs	15 (53.6)	14 (93.3)	**0.015**	11 (50)	16 (84.2)	**0.046**
Methotrexate	8 (28.6)	4 (26.6)	1.000	6 (27.3)	5 (26.3)	1.000
Azathioprine	7 (25)	5 (33.3)	0.723	5 (22.7)	7 (36.8)	0.493
Mycophenolate mofetil	0 (0)	3 (20)	**0.037**	0 (0)	3 (15.8)	0.091
Cyclophosphamide	0 (0)	2 (13.3)	0.116	0 (0)	1 (5.3)	0.463
Leflunomide	0 (0)	1 (6.6)	0.349	0	1 (5.3)	0.463
Biologic therapy						
Rituximab	3 (10.7)	5 (33.3)	0.069	1 (4.5)	7 (36.8)	**0.016**

Results are expressed in median (minimum and maximum values) and n (%). SC, Seroconversion defined as a positive serology (IgG titer ≥15 AU/mL) for anti-SARS-CoV-2 S1/S2 IgG antibodies after vaccination (Indirect ELISA, LIAISON® SARS-CoV-2 S1/S2 IgG, DiaSorin, Italy). Positivity for Nabs defined as a neutralizing activity ≥ 30% (cPass sVNT Kit, GenScript, Piscataway).

#### Six-months (D210) immunogenicity decay in 32 AAV and 32 CG groups after the second vaccine dose

Antibody decay in six months was observed with a trend of 15.7% reduction in IgG seropositivity for 32 AAV patients (68.8% vs. 53.1%, p = 0.074) and 18.7% for 32 GC (100% vs. 81.3%, p = 0.041). GMT titers also had a significant reduction of 39.2% in AAV patients (26.5 [14.9–46.9] vs. 16.1 [8.7–29.9], p = 0.010) and an even more striking decrease of 54.8% for the CG (83.7 [69.3–101.3] vs. 37.8 [25.0–57.2], p < 0.001). For the NAb positivity the 6-month decrease in the rate for the 32 AAV patients (59.4% vs. 40.6%, p = 0.114) was not significant, whereas for the CG a 62.5% reduction was observed in CG (90.6% vs. 28.1%, p < 0.001).

#### Booster dose immunogenicity in 32 AAV patients and 32 CG from D210 to D240

The booster dose resulted in a 21.9% increase in anti-SARS-CoV-2 IgG antibodies positivity in AAV patients (53.1% vs. 75%, p = 0.023) and 18.7% in CG (81.3% vs. 100%, p = 0.041). AAV patients remained lower than the CG group at D240 (75% vs. 100%, p = 0.005). A 4.3-fold augmentation in GMT was observed for the AAV group (16.1 [8.7‒29.9] vs. D240 70.0 [34.8‒140.7] p < 0.0001) and 6.3-fold for CG (37.8 [25.0‒57.2] vs. D240 237.8 [195.8‒288.6] p < 0.0001). For NAb positivity the same pattern was observed but with a more relevant increment of 34.4% in the AAV patients (40.6% vs. 75%, p = 0.006) and 68.8% in CG (28.1% vs. 96.9%, p < 0.0001). NAb activity increased 1.4-fold for AAV patients (59.2 [48.9‒75.3] vs. D240 82.1 [57.2‒95.7] p = 0.006) and no change was detected for the CG (86.3 [54.1‒95.0] vs. D240 82.5 [62.7‒97.1] p = 0.065)].

#### Vaccine tolerance and safety 53 AAV patients and 106 CG at D28 and D69

No serious adverse reaction was observed in both group and the events were similar between the former group and CG, except for the hospitalization of one patient in the AAV group on the date of his second vaccine dose, due to a urinary tract infection, which was not considered to be a vaccine-related adverse event. This patient was excluded from the study for not completing the vaccine protocol at the scheduled interval but had a complete recovery from the infection and subsequently completed the vaccination. After the first dose of CoronaVac, there was a higher prevalence of malaise (p = 0.007), myalgia (p = 0.021), and sneezing (p = 0.017) in AAV patients when compared with CG, and after the second dose, the events were similar the former group and CG (p = 0.696) (Supplementary Table 1). For booster dose, only mild AE was observed in 12 (37.5%) AAV patients and 6 (18.8%) CG (p = 0.111). There was 1 incident case of COVID-19 in an AAV patient during the study and 2 cases in the control group, all with mild symptoms and no need for hospitalization.

## Discussion

ANCA-associated vasculitis patients are among the high-risk groups of SARS-CoV-2 serious infection and death. The present results show that two doses of the inactivated CoronaVac had moderate immunogenicity in naïve AAV patients, lower than the control group. AAV patients had a mild decrease in humoral response in six months and a good response with a booster dose. Furthermore, the authors showed that this vaccine was safe in this group of patients.

The immunogenicity in AAV patients was moderate and the inclusion of only naïve patients may have influenced this finding. In fact, the authors have previously demonstrated that naïve ARD and COVID-19 pre-exposed ARD patients have distinct dynamics of vaccine response, with a significantly lower antibody production in the former group.^23^ In spite of that, naïve AAV had a lower response compared to naïve Systemic Lupus (SLE) patients (70.2%) immunized with the same vaccine and also reduced when compared to the naïve CG. For the healthy control group, age and sex, known relevant factors to impair vaccine response, is not the likely explanation since groups were balanced for these parameters. With regard to SLE, the older age and the distinct sex distribution of AAV may have contributed to the reduced vaccine-induced antibody response in these patients.^24^ In addition, other and the authors have demonstrated that the major factor to influence vaccine response is therapy and in fact, the frequency of methotrexate and rituximab was higher in AAV patients than in lupus.^9^^,^^11^ Unexpectedly, neutralizing activity of anti-SARS-CoV-2 antibodies was comparable to the control group, a reassuring finding since this parameter was reported to be a more precise maker of disease protection than anti-SARS-CoV-2 IgG positivity.^25^

Regarding factors that influence vaccine response, the authors observed herein, that the use of prednisone is a major contributor to the decreased immunogenicity in AAV patients. A similar finding was reported for mRNA and inactivated vaccines in general ARD patients,^9^^,^^11^^,^^26^^,^^27^ but none evaluated specifically AAV patients. Regarding the impact of drugs in the present study, mycophenolate mofetil appeared to reduce seroconversion, while rituximab impacted mainly NAb production (Table 5). Accordingly, rituximab and methotrexate, two medications used very often to treat AAV, were reported to decrease vaccine response.^28^, ^29^, ^30^ However, this impact was not confirmed in the multivariate analysis, probably due to the small representation of the patients under these therapies. Reinforcing this possibility, in the large CoronavRheum trial, of which the present study is part, the multivariate analysis revealed that methotrexate, mycophenolate, and rituximab had a deleterious effect on vaccine response.^11^

Immunogenicity waning is a major concern for these immunocompromised patients and a 65% reduction in IgG levels and 70% for neutralizing antibody concentrations in 6-months was reported for participants with immunosuppression immunized with mRNA vaccine.^31^ A lower reduction in IgG levels (38%) and neutralizing antibody activity (54%) was observed by the group for a large overall ARD population with 818 patients immunized with inactivated vaccine.^32^ The pattern of antibody decay for AAV was distinct with a non-significant reduction in NAb activity (15.1%) in 6-months.

The booster dose was effective in increasing seroconversion and NAb positivity in both groups 6-months after the first vaccine dose, reinforcing the recommendation of this strategy for this group of patients, as suggested by the Center of Disease Control.^33^ Anti-SARS-CoV-2 IgG positivity was, however, lower than observed in overall ARD patients after the third dose^34^ probably related to higher frequencies of patients under rituximab in the present study.

Importantly, the authors demonstrated that the vaccine was safe in this group of patients, with no serious adverse effects and a general safety profile compared with the control group. Duran and colleagues^35^ showed that infection by the SARS-CoV-2 has triggered a few cases of AAV in previously healthy individuals. The present data suggest that the vaccine does not worsen disease control in patients with pre-existing vasculitis. Another important observation was that disease activity, measured by the BVAS, did not seem to impact vaccine immunogenicity.

The main limitation of the present study is the small sample size related to the rarity of the disease and the lack of cellular immune response assessment.

In conclusion, this study provides novel data on moderate immunogenicity and an excellent safety profile of CoronaVac in AAV patients. A six-months mild antibody waning was observed with a good response to the booster dose, although levels remained lower than CG.

## Conflicts of interest

The authors declare no conflicts of interest.

## Appendix Supplementary materials

Image, application 1
