# Supplementary material for: Anti-SARS-CoV-2 inactivated vaccine in patients with ANCA-associated vasculitis: Immunogenicity, safety, antibody decay and the booster dose
Source: Clinics (Sao Paulo). 2022 Nov 29;78:100150. doi: 10.1016/j.clinsp.2022.100150 (PMC9705212; doi:10.1016/j.clinsp.2022.100150)
Supplement: Supplementary file 1 [file mmc1.docx]

CLINICS-D-22-00149_Supplementary Material

**Supplementary Table 1** Adverse events of CoronaVac vaccination in AAV patients and controls.

|  | **After vaccine 1^st^ dose** | | | **After vaccine 2^nd^ dose** | | |
| --- | --- | --- | --- | --- | --- | --- |
|  | **Vasculitis (n = 53)** | **Controls (n = 106)** | **p-value** | **Vasculitis (n = 42)** | **Controls (n = 89)** | **p-value** |
| **No symptoms** | 34 (64.1) | 65 (61.3) | 0.862 | 26 (61.9) | 30 (33.7) | **0.004** |
| **Local reactions** (at the injection site) | 7 (13.2) | 19 (17.9) | 0.503 | 6 (14.3) | 14 (15.7) | 1.000 |
| Pain | 6 (9.4) | 16 (15.1) | 0.630 | 5 (11.9) | 13 (14.6) | 0.790 |
| Erythema | 2 (3.8) | 5 (4.7) | 1.000 | 2 (3.7) | 3 (3.4) | 0.655 |
| Swelling | 1 (1.9) | 5 (4.7) | 0.664 | 0 (0) | 4 (4.5) | 0.305 |
| Bruise | 0 (0) | 2 (1.9) | 0.552 | 0 (1.2) | 1 (1.1) | 1.000 |
| Pruritus | 0 (0) | 2 (1.9) | 0.552 | 0 (3.7) | 4 (4.5) | 0.305 |
| Induration | 1 (1.9) | 3 (2.8) | 1.000 | 2 (2.5) | 3 (3.4) | 0.655 |
| **Systemic reactions** | 17 (32.1) | 33 (31.1) | 1.000 | 12 (32.1) | 27 (30.3) | 1.000 |
| Fever | 1 (1.9) | 2 (1.9) | 1.000 | 0 (1.2) | 4 (4.5) | 0.305 |
| Malaise | 7 (13.2) | 2 (1.9) | **0.007** | 4 (7.4) | 4 (4.5) | 0.268 |
| Somnolence | 4 (7.5) | 7 (6.6) | 1.000 | 5 (11.1) | 7 (7.9) | 0.521 |
| Lack of appetite | 1 (1.9) | 1 (0.9) | 1.000 | 2 (2.5) | 3 (3.4) | 0.655 |
| Nausea | 3 (5.7) | 1 (0.9) | 0.105 | 2 (3.7) | 6 (6.7) | 1.000 |
| Vomit | 0 (0) | 0 (0) | ‒ | 0 (0) | 2 (2.2) | 1.000 |
| Diarrhea | 2 (3.8) | 7 (6.6) | 0.719 | 3 (4.9) | 4 (4.5) | 0.682 |
| Abdominal pain | 3 (5.7) | 2 (1.9) | 0.334 | 2 (4.9) | 3 (3.4) | 0.655 |
| Vertigo | 4 (7.5) | 3 (2.8) | 0.223 | 3 (3.7) | 3 (3.4) | 0.391 |
| Tremor | 0 (0) | 0 (0) | ‒ | 0 (1.2) | 0 (0) | ‒ |
| Headache | 7 (13.2) | 9 (8.5) | 0.411 | 8 (13.6) | 16 (17.9) | 1.000 |
| Fatigue | 6 (11.3) | 5 (4.7) | 0.182 | 2 (9.9) | 10 (11.2) | 0.336 |
| Sweating | 2 (3.8) | 0 (0) | 0.110 | 1 (1.2) | 0 (0) | 0.321 |
| Myalgia | 8 (15.1) | 4 (3.8) | **0.021** | 4 (6.2) | 8 (9.0) | 1.000 |
| Muscle weakness | 5 (9.4) | 3 (2.8) | 0.118 | 3 (4.9) | 5 (5.6) | 0.715 |
| Arthralgia | 6 (11.3) | 5 (4.7) | 0.182 | 4 (7.4) | 5 (5.6) | 0.467 |
| Back pain | 3 (5.7) | 3 (2.8) | 0.411 | 5 (8.6) | 6 (6.7) | 0.329 |
| Cough | 2 (3.8) | 1 (0.9) | 0.258 | 1 (3.7) | 7 (7.9) | 0.435 |
| Sneezing | 7 (13.2) | 3 (2.8) | **0.016** | 4 (8.6) | 8 (9.0) | 1.000 |
| Coryza | 5 (9.4) | 9 (8.5) | 1.000 | 7 (11.1) | 7 (7.9) | 0.140 |
| Stuffy nose | 4 (7.5) | 3 (2.8) | 0.223 | 3 (4.9) | 1 (1.1) | 0.101 |
| Sore throat | 1 (1.9) | 5 (4.7) | 0.664 | 3 (4.9) | 3 (3.4) | 0.391 |
| Shortness of breath | 2 (3.8) | 1 (0.9) | 0.258 | 2 (3.7) | 3 (3.4) | 0.661 |
| Conjunctivitis | 2 (3.8) | 0 (0) | 0.110 | 0 (0) | 2 (2.2) | 1.000 |
| Pruritus | 2 (3.8) | 2 (1.8) | 0.601 | 1 (3.7) | 3 (3.4) | 1.000 |
| Skin rash | 1 (1.9) | 2 (1.8) | 1.000 | 1 (1.2) | 0 (0) | 0.321 |

Results are presented in n (%).
